# Patterns of Prescription Medication Use Before Diagnosis of Early Age-Onset Colorectal Cancer: Population-Based Descriptive Study

**DOI:** 10.2196/50402

**Published:** 2024-07-12

**Authors:** Vienna Cheng, Eric C Sayre, Vicki Cheng, Ria Garg, Sharlene Gill, Ameer Farooq, Mary A De Vera

**Affiliations:** 1 Faculty of Pharmaceutical Sciences University of British Columbia Vancouver, BC Canada; 2 Collaboration for Outcomes Research and Evaluation Faculty of Pharmaceutical Sciences University of British Columbia Vancouver, BC Canada; 3 British Columbia Centre on Substance Use Vancouver, BC Canada; 4 Leslie Dan Faculty of Pharmacy University of Toronto Toronto, ON Canada; 5 Faculty of Medicine University of British Columbia Vancouver, BC Canada; 6 BC Cancer Vancouver, BC Canada; 7 School of Medicine Division of General Surgery Queen’s University Kingston, ON Canada; 8 Centre for Health Evaluation and Outcome Sciences University of British Columbia Vancouver, BC Canada

**Keywords:** colorectal cancer, medications, medication patterns, cancer diagnosis, prediagnosis, prescriptions, patterns, early-onset, population-based, incidence, male individuals, female individuals, health databases, pharmacology, diagnostic, descriptive study, gastroenterology, cancers

## Abstract

**Background:**

Colorectal cancer (CRC) is estimated to be the fourth most common cancer diagnosis in Canada (except for nonmelanoma skin cancers) and the second and third leading cause of cancer-related death in male and female individuals, respectively.

**Objective:**

The rising incidence of early age-onset colorectal cancer (EAO-CRC; diagnosis at less than 50 years) calls for a better understanding of patients’ pathway to diagnosis. Therefore, we evaluated patterns of prescription medication use before EAO-CRC diagnosis.

**Methods:**

We used linked administrative health databases in British Columbia (BC), Canada, to identify individuals diagnosed with EAO-CRC between January 1, 2010, and December 31, 2016 (hereinafter referred to as “cases”), along with cancer-free controls (1:10), matched by age and sex. We identified all prescriptions dispensed from community pharmacies during the year prior to diagnosis and used the Anatomical Therapeutic Chemical Classification system Level 3 to group prescriptions according to the drug class. A parallel assessment was conducted for individuals diagnosed with average age-onset CRC (diagnosis at age 50 years and older).

**Results:**

We included 1001 EAO-CRC cases (n=450, 45% female participants; mean 41.0, SD 6.1 years), and 12,989 prescriptions were filled in the year before diagnosis by 797 (79.7%) individuals. Top-filled drugs were antidepressants (first; n=1698, 13.1%). Drugs for peptic ulcer disease and gastroesophageal reflux disease (third; n=795, 6.1%) were more likely filled by EAO-CRC cases than controls (odds ratio [OR] 1.4, 95% CI 1.2-1.7) and with more frequent fills (OR 1.8, 95% CI 1.7-1.9). We noted similar patterns for topical agents for hemorrhoids and anal fissures, which were more likely filled by EAO-CRC cases than controls (OR 7.4, 95% CI 5.8-9.4) and with more frequent fills (OR 15.6, 95% CI 13.1-18.6).

**Conclusions:**

We observed frequent prescription medication use in the year before diagnosis of EAO-CRC, including for drugs to treat commonly reported symptoms of EAO-CRC.

## Introduction

Colorectal cancer (CRC) is estimated to be the fourth most common cancer diagnosis in Canada (except for nonmelanoma skin cancers) and the second and third leading cause of cancer-related death in male and female individuals, respectively [1]. Given the marked onset of CRC among individuals aged 50 years, it was historically considered a disease for older adults. However, recent evidence particularly over the past decade has revealed a rise in the incidence of early age-onset CRC (EAO-CRC), defined as diagnosis among those younger than 50 years [2]. For example, a 2020 Canadian study [3] showed that between 2008 and 2017, the 30- to 39-year age group accounted for the most significant increase with age-specific average annual percent changes of 4.33 (95% CI 2.79-5.91) for female individuals and 4.53 (95% CI 2.89-6.19) for male individuals.

The increasing incidence of EAO-CRC has called for research to better understand various aspects of the disease [2-4], including the path to diagnosis, particularly patterns of health care use. Using administrative health databases in British Columbia (BC), Canada, a 2022 case-control study found that in comparison to age- and sex-matched cancer-free controls, individuals diagnosed with EAO-CRC experienced a marked increase in outpatient physician visits during the year prior to diagnosis, with the reason for visit most commonly documented as nausea, vomiting, and abdominal pain [5]. Therefore, delineating patterns of prescription medication use before diagnosis of EAO-CRC may provide further insight, particularly as certain pharmacologic treatments may suggest potential diagnostic opportunities for EAO-CRC. In 2017, Pottegård and Hallas [6] used the Danish Cancer Registry to evaluate prescription drug use in the 24 months preceding a diagnosis of lung, breast, colon, and prostate cancers and found a stable pattern that markedly increased at 6 months before diagnosis. Among a prespecified list of drug classes that may likely be prescribed for early symptoms of one of the cancers studied (eg, drugs against overactive bladder may be associated with future prostate cancer diagnosis and drugs against constipation or diarrhea may be associated with future colon cancer diagnosis), such as opioids, oral antidiabetics, and statins, authors found that for those with colon cancer, the increased prescription rates before diagnosis were for proton pump inhibitors and antibiotics [6]. It is important to assess whether a similar pattern is presenting in another jurisdiction with a specific focus on CRC and considering age at diagnosis, particularly given the increasing incidence of EAO-CRC [2-4]. Thus, our primary aim was to assess patterns of prescription medication use among individuals with EAO-CRC during the year preceding diagnosis. To contextualize our findings, we also assessed patterns of prescription medication use among age- and sex-matched cancer-free controls and individuals diagnosed with average-age onset CRC (AAO-CRC; 50 years and older). We aim to better understand the pathway to diagnosis through evaluating patterns of prescription medication use in the year preceding EAO-CRC diagnosis.

## Methods

### Data Sources

As with prior population-based research on the epidemiology of EAO-CRC [7], we linked administrative health databases capturing longitudinal and deidentified individual-level health services data for the province of BC, Canada [8-14]. Population Data BC facilitated data access to the Medical Services Plan database on outpatient visits [13], the Discharge Abstract Database on inpatient visits [14], the Consolidation File for demographics [11], the Vital Statistics File for deaths [12], and the PharmaNet database on all prescriptions dispensed in community pharmacies regardless of payer [15]. These databases were linked to the BC Cancer Registry, which includes data on cancer diagnosis (eg, date and site) [9].

### Study Design

A population-based descriptive observational study was conducted. First, we identified CRC cases as individuals diagnosed with CRC between January 1, 2010, and December 31, 2016, using *International Classification of Diseases for Oncology, Third Edition* (ICD-O-3) codes, specifically: C18.2-C18.9 (colon), C19.9 (rectosigmoid), and C20 and C21.8 (rectum). Our study period coincided with the beginning (in 2010) of population-based reporting of staging data, based on American Joint Committee on Cancer staging guidelines, with >85% capture in the BC Cancer Registry [16,17]. We assigned the *index date* as the date of definitive diagnosis from the BC Cancer Registry based on tissue diagnosis of CRC (endoscopist, surgeon, or oncologist). Next, we further classified cases as those with EAO-CRC (diagnosed at less than 50 years of age) and AAO-CRC (diagnosed at 50 years of age or later). We matched individuals with CRC to cancer-free controls (1:up to 10) on age and sex. Controls were also required to have a health care use (ie, outpatient visit, hospitalization, or prescription fill) within the same year their matched case was diagnosed. Controls were assigned an index date, which corresponded to their match date (Multimedia Appendix 1 illustrates data sources and study sample).

### Prescription Medication Use

We assessed the use of prescription medications over the 1-year period preceding the index date using the PharmaNet database. We drew rationale for evaluating the 1-year period before diagnosis from the study by Pottegård and Hallas [6] showing marked prescription drug use 6 months before cancer diagnosis and from our own prior work with patterns of outpatient physician visits the year before cancer diagnosis [5]. By law, prescriptions dispensed from community pharmacies in BC must be entered in PharmaNet, a province-wide network [15]. Thus, we were able to assess all prescriptions, regardless of payer, and extracted relevant information including prescription date, drug identification number, and Anatomical Therapeutic Chemical (ATC) classification [18]. In particular, we used the third-level ATC code, allowing us to categorize drugs according to first level—main anatomical or pharmacological group (eg, A alimentary tract and metabolism); second level—pharmacological or therapeutic subgroup (eg, A10 drugs used in diabetes); third and fourth levels—chemical, pharmacological, or therapeutic subgroup (eg, A10B blood glucose–lowering drugs and A10BA biguanides; Multimedia Appendix 2).

### Statistical Analysis

We used descriptive statistics (eg, mean and proportions) to characterize all individuals included in our study sample according to age, sex (female or male), socioeconomic status (determined using neighborhood income per person equivalent adjusted for household size), type of residence (rural vs urban, determined using Census Metropolitan Area or Census Agglomeration from geographical census data). For individuals with CRC, the cancer site using ICD-O-3 codes (eg, rectum, left colon, right colon, and transverse colon) and stage at diagnosis were also determined.

We assessed patterns of prescriptions among EAO-CRC cases overall and according to sex and stage age at diagnosis, reporting counts and proportions using both prescriptions and persons as units of analyses. Using logistic regression, we evaluated determinants of our outcome of having ≥1 prescription filled in the year before diagnosis among EAO-CRC cases. Potential determinants included age, sex, neighborhood income quintile, residence, cancer diagnosis site, and stage. We used a backward stepwise approach and retained the model variables based on statistical or clinical significance. We then compared patterns of prescription medications among EAO-CRC cases and controls, reporting counts, proportions, and odds ratios (ORs) and corresponding 95% CI, where relevant. We also compared patterns of prescription medications among EAO-CRC and AAO-CRC cases, reporting counts, proportions, ORs and corresponding 95% CIs, where relevant. We completed all these analyses using SAS statistical software (version 9.4; SAS Institute).

### Study Conduct

All inferences, opinions, and conclusions drawn in this paper are those of the authors and do not reflect the opinions or policies of the Data Stewards.

### Ethical Considerations

This study was approved by the University of British Columbia’s Behavioural Research Ethics Board (H17-03530) and was performed in accordance with relevant guidelines and regulations. Consent to participate was waived by the University of British Columbia’s Behavioural Research Ethics Board, as this research involves secondary use of data. Individual-level health services data from the linked administrative health databases were deidentified or scrambled.

## Results

Our study included 1001 cases with EAO-CRC (n=450, 45% female participants; mean age 41.0, SD 6.1 years) and 10,010 matched cancer-free controls (n=4500, 45% female participants; mean age 41.0, SD 6.1 years). As shown in Table 1, EAO-CRC cases were most frequently diagnosed with cancer in the rectum (n=418, 41.8%) and with stage III (n=351, 35%) and stage IV (n=270, 27%) disease. In our parallel analyses, we identified 12,331 cases with AAO-CRC (n=5536, 44.9% female participants, mean age 66.6, SD 9.2 years), who were most frequently diagnosed with cancer in the left colon (n=5210, 42.3%) and stage III (n=3644, 29.6%) or stage II (n=2996, 24.3%) disease.

There were 12,989 prescription events among 797 (79.7%) EAO-CRC cases and 174,806 prescription events among 7796 (77.9%) matched cancer-free controls. With respect to individuals, there is no significant difference in the proportions of EAO-CRC cases and controls filling prescriptions (OR 1.11, 95% CI 0.94-1.3). However, with respect to the number of prescriptions filled, among 797 EAO-CRC cases, there was a mean of 16.3 (SD 73.7) prescriptions (median 5.0) per case; whereas for 7796 controls, there was a mean of 22.4 (SD 99.3) prescriptions (median 6.0) per control. Multimedia Appendix 3 summarizes medication classes that represent ≥1% (n≥130 prescriptions for EAO-CRC cases and n≥1748 prescriptions for controls) of all prescriptions in the year before diagnosis for EAO-CRC cases and controls. Assessing specific medications including ranking and frequency revealed patterns of use. For example, antidepressants (ATC3 N06A) were the top medications filled by both EAO-CRC cases (n=1698, 13.1% of prescriptions) and controls (n=17,262, 9.9% of prescriptions) with EAO-CRC having more frequent fills (OR 1.4, 95% CI 1.3-1.4) than cases. Gastrointestinal drugs (ATC3 N02A; for peptic ulcer disease and gastroesophageal reflux disease) were the third most filled prescriptions by EAO-CRC cases (n=795, 6.1% of prescriptions) and fifth most filled by controls (n=6126, 3.5% of prescriptions) with EAO-CRC cases having higher odds of filling (OR 1.4, 95% CI 1.2-1.7) and having more frequent fills (OR 1.8, 95% CI 1.7-1.9). Relatedly, agents for the treatment of hemorrhoids and anal fissures for topical use (ATC3 C05A) and drugs for constipation (ATC3 A06A) represent the ninth (n=275, 2.1% of prescriptions) and tenth (n=250, 1.9% of prescriptions) most filled prescriptions by EAO-CRC cases, respectively, but were not among ≥1% (n≥1748 prescriptions) of prescriptions for controls. EAO-CRC cases had higher odds of filling (OR 7.4, 95% CI 5.8-9.4) and had more frequent fills (OR 15.6, 95% CI 13.1-18.6) for topical agents for hemorrhoids and anal fissures. Among EAO-CRC cases, factors associated with filling 1 or more prescriptions in the year before diagnosis included having inflammatory bowel disease (adjusted odds ratio [aOR] 3.43; 95% CI 1.20-9.78) and depression (aOR 4.20, 95% CI 1.49-11.85). As well, number of outpatient visits was also a determinant with an aOR of 1.14 (95% CI 1.09-1.18).

**Table 1:** Characteristics of individuals with EAO-CRC^a^ (less than 50 years), AAO-CRC^b^ (50 years and older), and their respective controls.

Characteristic			EAO-CRC					AAO-CRC		
			Cases (n=1001)		Controls (n=10,010)		Cases (n=12,331)			Controls^c^ (n=123,310)
Age (years), mean (SD)			41 (6.1)		41 (6.1)		66.6 (9.2)			66.6 (9.2)
Female participants, n (%)			450 (45)		4500 (45)		5536 (44.9)			55,360 (44.9)
**Neighborhood income quintile, n (%)**										
	Quintile 1	191 (19.5)		2178 (21.8)		2585 (21)			24,750 (20.1)	
	Quintile 2	202 (19.6)		2071 (20.7)		2409 (19.7)			24,748 (20.1)	
	Quintile 3	205 (20.3)		2024 (20.2)		2455 (19.9)			24,561 (19.9)	
	Quintile 4	230 (22.9)		1993 (19.9)		2487 (20.1)			24,093 (19.5)	
	Quintile 5	173 (17.8)		1744 (17.4)		2395 (19.4)			25,158 (20.4)	
**Residence, n (%)**										
	Urban	887 (88.6)		9070 (90.6)		10,530 (85.4)			106,516 (86.4)	
	Rural	114 (11.4)		940 (9.4)		1801 (14.6)			16,794 (13.6)	
**Cancer diagnosis site, n (%)**										
	Rectum	418 (41.8)		N/A^d^		3848 (31.2)			N/A	
	Left colon	410 (41)		N/A		5210 (42.3)			N/A	
	Right colon	102 (10.2)		N/A		2232 (18.1)			N/A	
	Transverse colon	55 (5.5)		N/A		753 (6.1)			N/A	
**Cancer diagnostic stage, n (%)**										
	IV	270 (27)		N/A		2340 (19)			N/A	
	III	351 (35)		N/A		3644 (29.6)			N/A	
	II	205 (20.5)		N/A		2996 (24.3)			N/A	
	I	143 (14.3)		N/A		2680 (21.7)			N/A	
	0	32 (3.2)		N/A		671 (5.4)			N/A	

^a^EAO-CRC: early age-onset colorectal cancer.

^b^AAO-CRC: average age-onset colorectal cancer.

^c^Cancer-free controls for individuals with AAO-CRC were not analyzed for study purposes but reported demographic characteristics for completeness.

^d^N/A: not applicable.

We further assessed patterns of prescription medication use among EAO-CRC cases stratified by sex and stage. Multimedia Appendix 4 shows medication classes that represent ≥1% (n≥130 prescriptions) of all prescription events in the year before EAO-CRC diagnosis according to sex. We observed a higher number of prescriptions (n=7295) representing 56.2% of all events among 420/551 (76.2%) male EAO-CRC cases. In contrast, 377/450 (83.8%) female EAO-CRC cases had a lower number of prescriptions (n=5694) representing 43.8% of events. In terms of frequency of prescriptions by sex, we found higher fills for antidepressants (n=1075, 14.7% male patients and n=623, 10.9% female patients), antiepileptics (n=711, 9.8% male patients and n=421, 7.4% female patients), gastrointestinal drugs (n=582, 8% male patients and n=213, 3.7% female patients), as well as pain-related medications such as opioids (n=426, 5.8% male patients and n=219, 3.9% female patients) and other analgesics and antipyretics (n=79, 1.1% male patients and n=33, <1% female patients) for male patients with EAO-CRC than female patients with EAO-CRC. When EAO-CRC cases were stratified by stage, we observed the following prescription events among individuals: stage I (1620 prescription events in 112, 78.3% cases), stage II (3523 prescription events in 167, 81.5% cases), stage III (3226 prescription events in 283, 80.6% cases), and stage IV (4620 prescription events in 209, 77.4% cases). As seen visually by the blue bars in Multimedia Appendix 5, drugs belonging to the nervous system class were the most represented across all 4 stages. Of note, when considering number of prescriptions, those among stage IV EAO-CRC cases represented 35.6% (n=4620) of all prescription events in contrast to those among stage I EAO-CRC cases, which represented 12.5% (n=1620) of all prescription events. Antidepressants were the most filled medications among individuals diagnosed at stage II (n=502, 14.2%) and IV (n=880, 19%; Multimedia Appendix 5). Of interest, gastrointestinal drugs were the most used in stage IV EAO-CRC cases (n=510, 11%). Topical agents for the treatment of hemorrhoids and anal fissures were mostly filled by stage III EAO-CRC cases (n=128, 4%). Drugs for constipation were the highest used in stage II EAO-CRC cases (n=92, 2.6%) and lowest in stage I EAO-CRC cases (n=22, 1.4%).

For further context, when we analyzed 12,331 AAO-CRC cases, we observed a total of 317,271 prescription events among 10,979 (89%) individuals (Multimedia Appendix 6), mean of 28.9 (SD 83.9) prescriptions (median 13.0) per AAO-CRC case. While antidepressants (n=1698, 13.1%) and antiepileptics (n=1132, 8.7%) were the top 2 most frequently filled medications among EAO-CRC cases, these drug classes were observed to be the third and seventh most used medications among AAO-CCRC cases (n=15,097, 4.8% and n=10,689, 3.4%, respectively). Instead, the AAO-CRC group showed lipid modifying agents (n=21,898, 6.9%) and angiotensin-converting enzyme inhibitors (n=16,292, 5.1%) as the top 2 most used medication classes. Drugs that may be used to treat symptoms associated with potential CRC diagnosis were more frequently filled among EAO-CRC cases than AAO-CRC cases including gastrointestinal drugs (EAO-CRC: n=795, 6.1% and AAO-CRC: n=14,964, 4.7%), nonsteroidal anti-inflammatory and antirheumatic products (EAO-CRC: n=449, 3.5% and AAO-CRC: n=3430, 1.1%), topical agents for treatment of hemorrhoids and anal fissures (EAO-CRC: n=275, 2.1% and AAO-CRC: n=1672, <1%), and drugs for constipation (EAO-CRC: n=250, 1.9% and n=2897, <1%). EAO-CRC cases also revealed a higher use of opioids (EAO-CRC: n=645, 5% and AAO-CRC: n=9602, 3%).

## Discussion

### Overview

Using population-based administrative data, we assessed patterns of prescription medications in the year before diagnosis among individuals with EAO-CRC to understand the role of medications in the pathway to diagnosis in a condition that has seen a considerable increase in incidence [2-4]. Among 1001 EAO-CRC cases, 12,989 prescriptions were filled in the year before diagnosis by 797 (79.7%) individuals. With respect to medications, antidepressants were most commonly filled (n=1698, 13.1%), followed by antiepileptics (n=1132, 8.7%) and gastrointestinal drugs (ie, drugs for peptic ulcer disease and gastroesophageal reflux disease; n=795, 6.1%). Sex-based analyses revealed that male EAO-CRC cases had a higher number of prescriptions (n=7295, 56.2% of prescription events) but at a lower proportion (420/551, 76.2%), whereas female EAO-CRC cases had a lower number of prescriptions (n=5694, 43.8% of prescription events) but at a higher proportion (377/450, 83.8%).

### Principal Findings and Comparison to Prior Work

Given the increasing risk of EAO-CRC [4] and reported diagnostic delays in prior studies [19,20], we were particularly interested in studying the patterns of prescription medication use leading to diagnosis in individuals with EAO-CRC and understanding potential diagnostic opportunities. To our knowledge, this study is the first to assess patterns of prescription use before diagnosis of EAO-CRC. In 2017, using Danish nationwide health registries on cancer and prescription drugs, Pottegård and Hallas [6] assessed the new use of prescription drugs among patients with lung, breast, colon, and prostate cancers 24 months preceding their cancer diagnosis. Authors found similar patterns of drug use between cancer cases and population controls in the 24- to 12-month period before cancer diagnosis. Among colon cancer cases, authors showed an increase in the use of prespecified drug classes that were likely prescribed for symptoms relating to their cancer, namely, proton pump inhibitors, laxatives or drugs against diarrhea, and opioid analgesics. However, this study did not characterize participants, and as such, it is not feasible to draw findings according to age as well as sex and stage, as with our study. With respect to prescription medication use specifically among individuals with CRC, a 2021 cohort study by Engeland et al [21] using data from the Cancer Registry of Norway primarily assessed prescription medications after diagnosis but also reported on use in the year before diagnosis. Authors evaluated a prespecified list of drugs according to 5 major categories and reported the top 3 most commonly used drug groups in the year before diagnosis such as those for cardiovascular diseases (use prevalence 24.8%); endocrine, nutritional, and metabolic diseases (use prevalence 17.8%); and mental and behavioral disorders (use prevalence 6.7%). Although the study included patients with CRC aged 20-84 years, there was no reporting of drug use according to age groups. Furthermore, with 530 individuals in the 20- to 39-year age category comprising 2% of the study population, reported findings largely reflect drug use among older patients with CRC.

Indeed, this study provides a better understanding of patterns of prescription medication use specifically in EAO-CRC. In contrast to the aforementioned studies [6,21], which assessed prespecified lists of drugs based on reimbursement, we were able to assess all prescriptions, regardless of payer, given comprehensive capture in the PharmaNet database. At the outset, we initially assumed that the most common prescriptions filled during the year of diagnosis were for gastrointestinal and pain, based on previously reported symptoms of EAO-CRC [22]. Indeed, among the top 10 classes of most frequently filled prescriptions by EAO-CRC cases were gastrointestinal drugs for peptic ulcer disease and gastroesophageal reflux disease (third), opioids (fourth), anti-inflammatory and antirheumatic drugs and nonsteroids (sixth), topical agents for hemorrhoids and anal fissures (ninth), and drugs for constipation (10th). We believe the increased use of these drugs for EAO-CRC symptoms in the year prior to diagnosis may be the early manifestations of red flag signs and symptoms of CRC. A 2023 population-based case-control study by Fritz et al [23] identified 4 red-flag signs and symptoms (rectal bleeding, abdominal pain, diarrhea, and iron-deficiency anemia) that were associated with a heightened risk of EAO-CRC between 3 months to 2 years preceding diagnosis (ORs range between 1.34 and 5.13). These red flag symptoms align with the clinical indications of our results, where gastrointestinal drugs, pain medications, and rectal medications were among the top 10 classes of most frequently filled prescriptions by EAO-CRC cases in the year prior to diagnosis. These results highlight the importance of ensuring individuals younger than 50 years consistently presenting with these early warning signs, and symptoms or medication use patterns are being given ample opportunities for further work-up and early detection of CRC at their health care interactions. Stratified analyses by sex and stage further reveal patterns such as higher use of pain-related medications and gastrointestinal drugs by male EAO-CRC cases. Our findings also suggest sex differences in health care use in terms of more frequent prescriptions among a smaller number of male EAO-CRC cases compared to less frequent prescriptions among a greater number of female EAO-CRC cases. With respect to stage, gastrointestinal drugs were most used in stage IV EAO-CRC cases, topical agents for treatment of hemorrhoids and anal fissures were by stage III EAO-CRC cases, and drugs for constipation were the highest used in stage II EAO-CRC cases and lowest in stage IV EAO-CRC cases. In contextualizing findings with those of controls, while opioids (fourth), gastrointestinal drugs (fifth), and nonsteroidal anti-inflammatory and antirheumatic drugs (seventh) were among the top 10 classes of filled prescriptions by matched cancer-free controls, they were at a lower frequency than EAO-CRC cases. Interestingly, topical agents for hemorrhoids and anal fissures and drugs for constipation were not among ≥1% (n≥1748 prescriptions) of prescription events among controls.

Our findings on patterns of prescription medication use before diagnosis support a study rationale of exploring targets for raised awareness and education on the increasing risk of EAO-CRC to allied health care providers, particularly pharmacists. With patients reportedly seeing pharmacists 1.5 to 10 times more frequently than primary care physicians [24], these may represent windows of opportunity for education or identification of risks for diseases, including cancer. A survey of community pharmacists suggests that patients have long sought advice from pharmacists about possible cancer signs and symptoms [25]. With respect to CRC, pharmacists are gaining recognition for their roles in the initiation of average age screening in various jurisdictions [26-28]. In the United States, a 2-phased study showed high satisfaction among individuals from limited-income populations with pharmacists speaking to them regarding CRC screening [27]. In Spain, evaluation of a population-based CRC screening program showed high adherence by participating pharmacies (82.4%) with respect to distributing fecal immunochemical test kits and a high return rate by invitees (93.5%), demonstrating the important role that pharmacists play in the program [29]. There is indeed potential to expand on pharmacists’ roles when it comes to educating individuals regarding CRC, including younger adults about EAO-CRC. To date, calls to action have largely focused on increasing awareness among primary care physicians on the increasing risk of EAO-CRC [30,31]; however, it is also important to consider other health care providers, particularly pharmacists, given their accessibility and as prescriptions represent a frequent health care encounter prior to CRC diagnosis.

Aside from patterns of prescription medication use, a noteworthy finding from this study is that antidepressants represent the top prescribed drug class for EAO-CRC cases in the year before diagnosis, representing 13.1% (n=1698) of all prescription events. For context, antidepressants were also the top prescribed drug class for matched cancer-free controls but at a lower frequency, 9.9% (n=17,262). For further context, among AAO-CRC cases, antidepressants were the third most prescribed drug class (n=15,097, 4.8%) after angiotensin-converting enzyme inhibitors (n=16,292, 5.1%) and lipid modifying agents (n=21,898, 6.9%). A potential reason for this finding is a diagnostic delay of CRC that commonly occurs in the young patient population [20], which may lead to anxiety and depressive symptoms [32]. A systematic review that compared the delays and outcomes between younger and older patients with CRC found that younger patients are at a higher risk of experiencing delays from symptom onset to presentation, as they are not eligible for screening [20]. Consequently, a delay in cancer diagnosis in the younger population is associated with an increased risk of anxiety and depression [32]. A cross-sectional study in 2022 found that patient intervals (symptom onset to first seeing a general practitioner) of ≥1 month were associated with greater depression (aOR 1.7, 95% CI 1.1-2.5) compared to <1 month and having ≥3 prereferral general practitioner consultations were associated with greater anxiety (aOR 1.6, 95% CI 1.1-2.3) compared to 1-2 consultations [32]. The main reasons that could contribute to the increased risk of emotional distress in the adolescents and young adult population prior to a diagnosis include patients’ persistent symptoms being dismissed due to young age, unresolved symptoms, and the fear of a potential cancerous diagnosis [32,33]. Furthermore, a 2022 cohort study that used the same administrative databases as this study found that compared to individuals without cancer, those with EAO-CRC did not have a higher onset of depression after diagnosis (adjusted hazard ratio [aHR] 1.00, 95% CI 0.92-1.10) [34]. However, individuals with EAO-CRC had a 41% higher risk of onset of depression after diagnosis compared to individuals with AAO-CRC (aHR 1.41, 95% CI 1.25-1.60) [34]. Since we were not able to link indications to prescription events, we do not know whether antidepressants were prescribed for depression or for other reasons, such as pain. Nonetheless, findings in this study suggest a substantial burden of depression even before EAO-CRC diagnosis, which further indicates the need for person-centered mental health services for individuals with EAO-CRC across the entire spectrum of care.

### Strengths and Limitations

The strengths and limitations of this study warrant discussion. We drew EAO-CRC cases and controls from population-based administrative health databases, namely Population Data BC and the BC Cancer Registry, which capture data on approximately 95% of all cancer cases in the province [9]. The BC Cancer Registry is reviewed annually for quality, completeness, and accuracy by the North American Association of Central Cancer Registries [9]. Nevertheless, this study is vulnerable to inherent limitations with administrative health data, which are not collected for research purposes. Although we have data on cancer stage, it is important to note this information in the BC Cancer Registry is not acquired using a systematic approach with sources including death certificates, pathology reports, and death certificates. Finally, administrative databases in BC do not yet capture information on the social construct of gender, and as such, we are not able to incorporate this into our analysis.

### Conclusions and Future Directions

Altogether, using generalizable, population-based data, including a complete capture of all prescriptions, we delineated patterns of medication use before diagnosis of EAO-CRC. Our findings suggest a high frequency of prescription fills in the year before diagnosis of EAO-CRC, including for drugs to treat commonly reported symptoms of EAO-CRC. As efforts continue to raise awareness on the increasing risk of EAO-CRC, our findings provide support for also considering the role of other health care providers, particularly pharmacists. Altogether, prescription medications represent a common and potentially, frequent, point-of-contact with the health care system and thus may lend to a better understanding of trajectories for individuals with EAO-CRC.

## Appendix

Multimedia Appendix 1 Overview of data sources and study sample (dashed arrow indicates linkages between databases using personal health numbers, which are then deidentified or scrambled).

Multimedia Appendix 2 Anatomical Therapeutic Chemical Level 1 groups.

Multimedia Appendix 3 Frequency of prescriptions in the year before diagnosis for early age-onset colorectal cancer cases and cancer-free controls according to Anatomical Therapeutic Chemical Level 3 Classification.

Multimedia Appendix 4 Frequency of prescriptions in the year before diagnosis among male and female early age-onset colorectal cancer cases, according to Anatomical Therapeutic Chemical Level 3 Classification.

Multimedia Appendix 5 Bar charts showing percentage of prescriptions for the top 10 drug classes by Anatomical Therapeutic Chemical Classification Level 3 code, according to stage for early age-onset colorectal cancer cases.

Multimedia Appendix 6 Frequency of prescriptions in the year before diagnosis for early age-onset colorectal cancer and average age-onset colorectal cancer cases according to Anatomical Therapeutic Chemical Level 3 Classification.

## Abbreviations

AAO-CRC: average age-onset colorectal cancer
aHR: adjusted hazard ratio
aOR: adjusted odds ratio
ATC: Anatomical Therapeutic Chemical
BC: British Columbia
CRC: colorectal cancer
EAO-CRC: early age-onset colorectal cancer
ICD-O-3: International Classification of Diseases for Oncology, Third Edition
OR: odds ratio

## Data Availability

The data that support the findings of this study are available from Population Data BC but restrictions apply to the availability of these data, which were used under license for this study, and so are not publicly available. Data are available from Population Data BC through a data access request. Therefore, the data sets generated and analyzed during this study are not publicly available due to strict data sharing agreements with the BC Ministry of Health but are available from the corresponding author on reasonable request.
