# Supplementary material for: Patterns of Prescription Medication Use Before Diagnosis of Early Age-Onset Colorectal Cancer: Population-Based Descriptive Study
Source: JMIR Cancer. 2024 Jul 12;10:e50402. doi: 10.2196/50402 (PMC11282380; doi:10.2196/50402)
Supplement: Multimedia Appendix 1 [file cancer_v10i1e50402_app1.pdf]

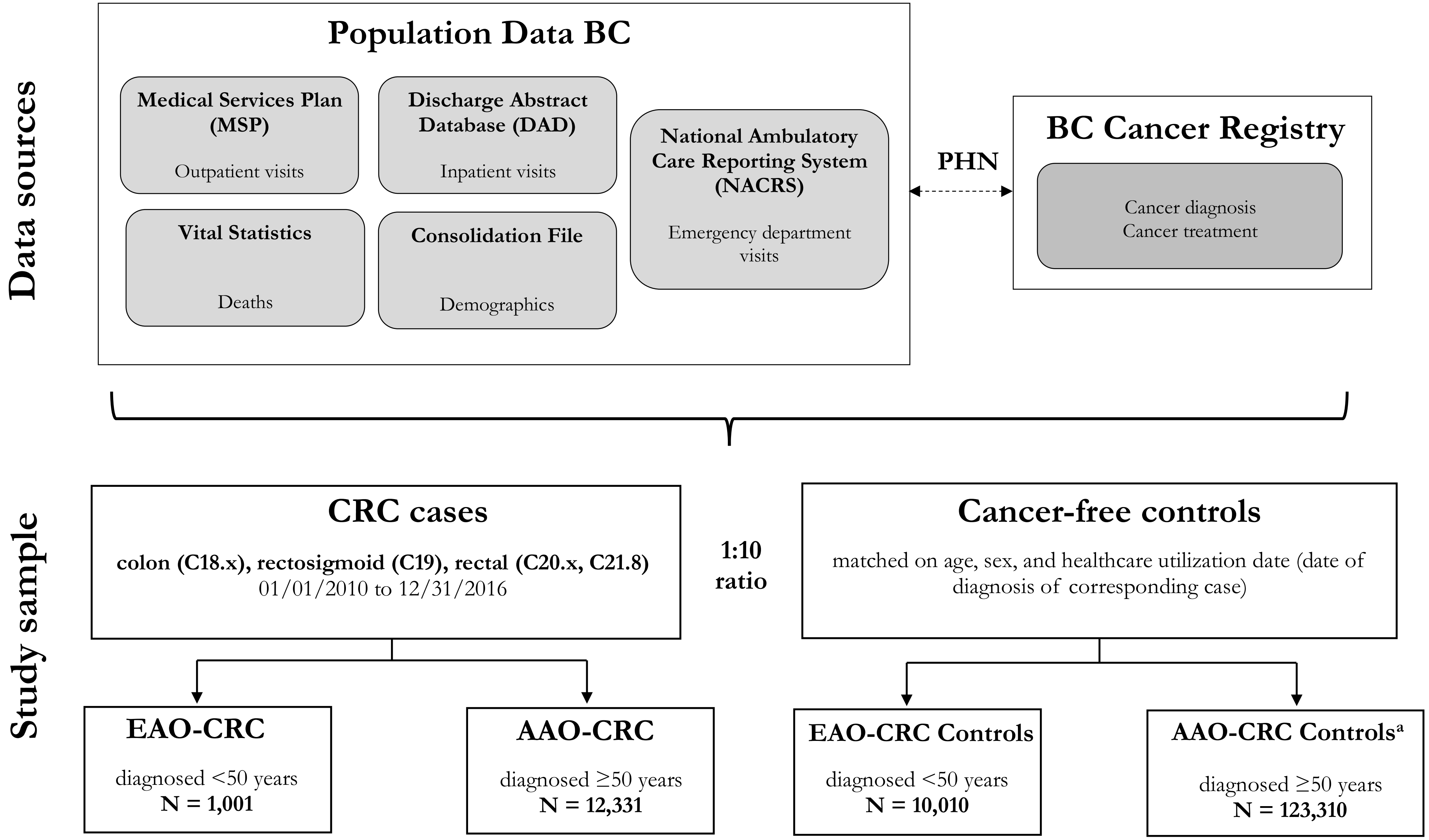

[a]Cancer-free controls for individuals with AAO-CRC were not analyzed for study purposes but reporting demographic characteristics for completeness.
