# Supplementary material for: Patterns of Prescription Medication Use Before Diagnosis of Early Age-Onset Colorectal Cancer: Population-Based Descriptive Study
Source: JMIR Cancer. 2024 Jul 12;10:e50402. doi: 10.2196/50402 (PMC11282380; doi:10.2196/50402)
Supplement: Multimedia Appendix 2 [file cancer_v10i1e50402_app2.docx]

| **ATC Level 1** | **Group Name** |
| --- | --- |
| **A** | Alimentary tract and metabolism |
| **B** | Blood and blood forming organs |
| **C** | Cardiovascular system |
| **D** | Dermatologicals |
| **G** | Genitourinary system and sex hormones |
| **H** | Systemic hormonal preparations, excluding sex hormones and insulins |
| **J** | Anti-infective for systemic use |
| **L** | Antineoplastic and immunomodulating agents |
| **M** | Musculoskeletal system |
| **N** | Nervous system |
| **P** | Antiparasitic products, insecticides and repellents |
| **R** | Respiratory system |
| **S** | Sensory organs |
| **V** | Various |
