# Supplementary material for: Patterns of Prescription Medication Use Before Diagnosis of Early Age-Onset Colorectal Cancer: Population-Based Descriptive Study
Source: JMIR Cancer. 2024 Jul 12;10:e50402. doi: 10.2196/50402 (PMC11282380; doi:10.2196/50402)
Supplement: Multimedia Appendix 3 [file cancer_v10i1e50402_app3.docx]

| **EAO-CRC Cases** | | | |  | **Controls** | | | |
| --- | --- | --- | --- | --- | --- | --- | --- | --- |
| **ATC Code** | **ATC 3 Class** | **Rx (n, %)** | **Persons (n, %)** |  | **ATC Code** | **ATC 3 Class** | **Rx (n, %)** | **Persons (n, %)** |
| **N06A** | Antidepressants | 1,698 (13.1) | 111 (11.1) |  | **N06A** | Antidepressants | 17,262 (9.9) | 1,478 (14.8) |
| **N03A** | Antiepileptics | 1,132 (8.7) | 60 (6.0) |  | **N05A** | Antipsychotics | 15,622 (8.9) | 510 (5.1) |
| **A02B** | GI drugs^a^ | 795 (6.1) | 150 (15.0) |  | **N03A** | Antiepileptics | 11,088 (6.3) | 696 (7.0) |
| **N02A** | Opioids | 645 (5.0) | 171 (17.1) |  | **N02A** | Opioids | 7,245 (4.1) | 1,718 (17.2) |
| **N05A** | Antipsychotics | 555 (4.3) | 30 (3.0) |  | **A02B** | GI drugs^a^ | 6,126 (3.5) | 1,098 (11.0) |
| **M01A** | Anti-inflammatory and antirheumatic products, non-steroids | 449 (3.5) | 133 (13.3) |  | **N05B** | Anxiolytics | 4,905 (2.8) | 867 (8.7) |
| **H03A** | Thyroid preparations | 344 (2.6) | 54 (5.4) |  | **M01A** | Anti-inflammatory and antirheumatic products, non-steroids | 4,653 (2.7) | 1,620 (16.2) |
| **N05B** | Anxiolytics | 340 (2.6) | 78 (7.8) |  | **N05C** | Hypnotics and sedatives | 4,128 (2.4) | 587 (5.9) |
| **C05A** | Agents for treatment of hemorrhoids and anal fissures for topical use | 275 (2.1) | 119 (11.9) |  | **N07B** | Drugs used in addictive disorders | 3,541 (2.0) | 404 (4.0) |
| **A06A** | Drugs for constipation | 250 (1.9) | 40 (4.0) |  | **A10B** | Blood glucose lowering drugs, excl. Insulins | 3,048 (1.7) | 340 (3.4) |
| **C09A** | Angiotensin-converting enzyme inhibitors, plain | 247 (1.9) | 38 (3.8) |  | **C10A** | Lipid modifying agents, plain | 2,839 (1.6) | 530 (5.3) |
| **N05C** | Hypnotics and sedatives | 207 (1.6) | 51 (5.1) |  | **H03A** | Thyroid preparations | 2,555 (1.5) | 506 (5.1) |
| **C10A** | Lipid modifying agents, plain | 199 (1.5) | 44 (4.4) |  | **C07A** | Beta blocking agents | 2,371 (1.4) | 338 (3.4) |
| **J01M** | Quinolone antibacterials | 199 (1.5) | 138 (13.8) |  | **M03B** | Muscle relaxants, centrally acting agents | 2,361 (1.4) | 607 (6.1) |
| **J01X** | Other antibacterials | 185 (1.4) | 146 (14.6) |  | **C09A** | Angiotensin-converting enzyme inhibitors, plain | 2,288 (1.3) | 463 (4.6) |
| **G03A** | Hormonal contraceptives for systemic use | 181 (1.4) | 49 (4.9) |  | **J01C** | Beta-lactam antibacterials, penicillins | 2,077 (1.2) | 1,538 (15.4) |
| **N07B** | Drugs used in addictive disorders | 174 (1.3) | 30 (3.0) |  | **R03A** | Adrenergics, inhalants | 2,009 (1.1) | 689 (6.9) |
| **R03A** | Adrenergics, inhalants | 172 (1.3) | 61 (6.1) |  | **--** | **--** | **--** | **--** |
| **B03A** | Iron preparations | 157 (1.2) | 35 (3.5) |  | **--** | **--** | **--** | **--** |
| **A10B** | Blood glucose lowering drugs, excl. Insulins | 154 (1.2) | 26 (2.6) |  | **--** | **--** | **--** | **--** |
| **J01C** | Beta-lactam antibacterials, penicillins | 141 (1.1) | 113 (11.3) |  | **--** | **--** | **--** | **--** |
| **A10A** | Insulins and analogues | 132 (1.0) | 14 (1.4) |  | **--** | **--** | **--** | **--** |
| **L04A** | Immunosuppressants | 130 (1.0) | 17 (1.7) |  | **--** | **--** | **--** | **--** |

^a^Gastrointestinal (GI) system drugs: drugs for peptic ulcer and gastro-oesophageal reflux disease
