# Supplementary material for: Patterns of Prescription Medication Use Before Diagnosis of Early Age-Onset Colorectal Cancer: Population-Based Descriptive Study
Source: JMIR Cancer. 2024 Jul 12;10:e50402. doi: 10.2196/50402 (PMC11282380; doi:10.2196/50402)
Supplement: Multimedia Appendix 4 [file cancer_v10i1e50402_app4.docx]

| **Male EAO-CRC** | | | | | **Female EAO-CRC** | | | | |
| --- | --- | --- | --- | --- | --- | --- | --- | --- | --- |
| **ATC Code** | | **ATC 3 Class** | **Rx (n, %)** | **Persons (n, %)** | **ATC Code** | **ATC 3 Class** | **Rx (n, %)** | **Persons (n, %)** | |
| **N06A** | | Antidepressants | 1,075 (14.7) | 44 (8.0) | **N06A** | Antidepressants | 623 (10.9) | 67 (14.9) | |
| **N03A** | | Antiepileptics | 711 (9.8) | 36 (6.5) | **N03A** | Antiepileptics | 421 (7.4) | 24 (5.3) | |
| **A02B** | | GI drugs^a^ | 582 (8.0) | 83 (15.1) | **H03A** | Thyroid preparations | 285 (5.0) | 45 (10.0) | |
| **N02A** | | Opioids | 426 (5.8) | 89 (16.2) | **N05A** | Antipsychotics | 263 (4.6) | 13 (2.9) | |
| **M01A** | | Anti-inflammatory & antirheumatic drugs, non-steroids | 351 (4.8) | 76 (13.8) | **N02A** | Opioids | 219 (3.9) | 82 (18.2) | |
| **N05A** | | Antipsychotics | 292 (4.0) | 17 (3.1) | **A02B** | GI drugs^a^ | 213 (3.7) | 67 (14.9) | |
| **C05A** | | Topical agents for hemorrhoids and anal fissures | 176 (2.4) | 67 (12.2) | **G03A** | Systemic hormonal contraceptives | 181 (3.2) | 49 (10.9) | |
| **N05B** | | Anxiolytics | 176 (2.4) | 33 (6.0) | **N05C** | Hypnotics & sedatives | 165 (2.9) | 33 (7.3) | |
| **A06A** | | Drugs for constipation | 158 (2.2) | 18 (3.3) | **N05B** | Anxiolytics | 164 (2.9) | 45 (10.0) | |
| **C09A** | | Angiotensin-converting enzyme inhibitors | 149 (2.0) | 28 (5.1) | **J01X** | Other antibacterials | 115 (2.0) | 89 (19.8) | |
| **C10A** | | Lipid modifying agents | 118 (1.6) | 33 (6.0) | **A11A** | Multivitamins, combinations | 104 (1.8) | . | |
| **R03A** | | Adrenergics, inhalants | 111 (1.5) | 31 (5.6) | **C05A** | Topical agents for hemorrhoids and anal fissures | 99 (1.7) | 52 (11.6) | |
| **A10B** | | Blood glucose lowering drugs, excluding insulin | 106 (1.5) | 17 (3.1) | **J01M** | Quinolone antibacterials | 99 (1.7) | 67 (14.9) | |
| **J01M** | | Quinolone antibacterials | 100 (1.4) | 71 (12.9) | **C09A** | Angiotensin-converting enzyme inhibitors | 98 (1.7) | 10 (2.2) | |
| **N07B** | | Drugs used for addictive disorders | 100 (1.4) | 11 (2.0) | **M01A** | Anti-inflammatory & antirheumatic drugs, non-steroids | 98 (1.7) | 57 (12.7) | |
| **C07A** | | Beta blocking agents | 86 (1.2) | 13 (2.4) | **B03A** | Iron preparations | 95 (1.7) | 18 (4.0) | |
| **N02B** | | Other analgesics and antipyretics | 79 (1.1) | 22 (4.0) | **A06A** | Drugs for constipation | 92 (1.6) | 22 (4.9) | |
| **A10A** | | Insulins and analogues | 76 (1.0) | 8 (1.5) | **R06A** | Systemic antihistamines | 92 (1.6) | 11 (2.4) | |
| -- | | -- | -- | -- | **C10A** | Lipid modifying agents | 81 (1.4) | 11 (2.4) | |
| -- | | -- | -- | -- | **J01C** | Beta-lactam antibacterials, penicillins | 75 (1.3) | 59 (13.1) | |
| -- | | -- | -- | -- | **N07B** | Drugs used for addictive disorders | 74 (1.3) | 19 (4.2) | |
| -- | | -- | -- | -- | **G03C** | Estrogens | 67 (1.2) | 7 (1.6) | |
| -- | | -- | -- | -- | **R03A** | Adrenergics, inhalants | 61 (1.1) | 30 (6.7) | |
| -- | | -- | -- | -- | **L04A** | Immunosuppressants | 60 (1.1) | 8 (1.8) | |
| ^a^Gastrointestinal (GI) system drugs: drugs for peptic ulcer and gastro-oesophageal reflux disease | | | | | | | |  |  |
