# Supplementary material for: Patterns of Prescription Medication Use Before Diagnosis of Early Age-Onset Colorectal Cancer: Population-Based Descriptive Study
Source: JMIR Cancer. 2024 Jul 12;10:e50402. doi: 10.2196/50402 (PMC11282380; doi:10.2196/50402)
Supplement: Multimedia Appendix 5 [file cancer_v10i1e50402_app5.pdf]

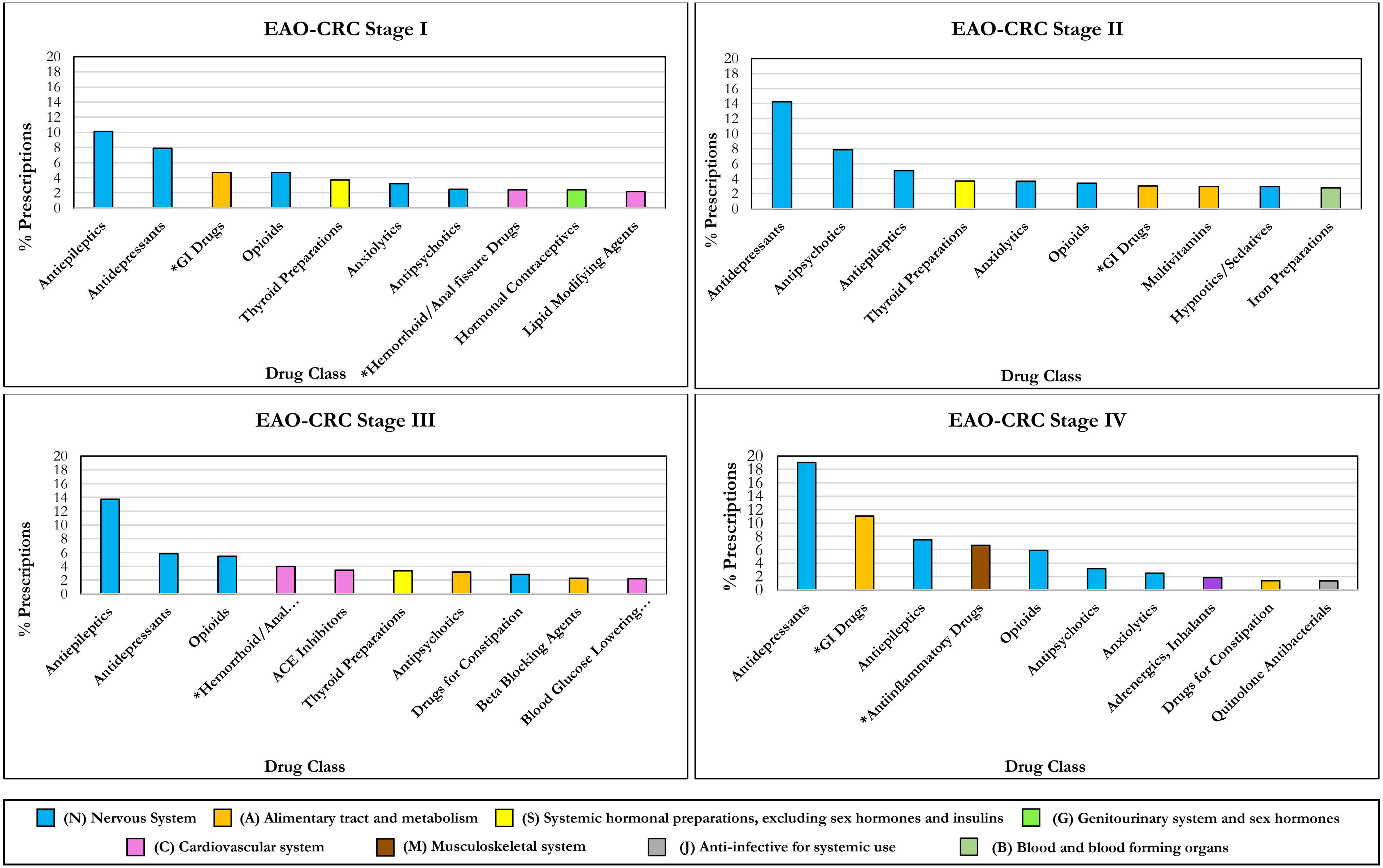

*Gastrointestinal (GI) system drugs: drugs for peptic ulcer and gastro-oesophageal reflux disease
*Hemorrhoid and anal fissure drugs: Agents for treatment of hemorrhoids and anal fissures for topical use
*Anti-inflammatory Drugs: Anti-inflammatory & Antirheumatic products, non-steroids
