# Supplementary material for: Patterns of Prescription Medication Use Before Diagnosis of Early Age-Onset Colorectal Cancer: Population-Based Descriptive Study
Source: JMIR Cancer. 2024 Jul 12;10:e50402. doi: 10.2196/50402 (PMC11282380; doi:10.2196/50402)
Supplement: Multimedia Appendix 6 [file cancer_v10i1e50402_app6.docx]

| **EAO-CRC cases** | | | |  | **AAO-CRC cases** | | | |
| --- | --- | --- | --- | --- | --- | --- | --- | --- |
| **ATC 3 Code** | **ATC 3 Class** | **Rx (n, %)** | **Persons (n, %)** |  | **ATC 3 Code** | **ATC 3 Class** | **Rx (n, %)** | **Persons (n, %)** |
| **N06A** | Antidepressants | 1,698 (13.1) | 111 (11.1) |  | **C10A** | Lipid modifying agents, plain | 21,898 (6.9) | 3,727 (30.2) |
| **N03A** | Antiepileptics | 1,132 (8.7) | 60 (6.0) |  | **C09A** | Angiotensin-converting enzyme inhibitors, plain | 16,292 (5.1) | 2,709 (22.0) |
| **A02B** | GI drugs^a^ | 795 (6.1) | 150 (15.0) |  | **N06A** | Antidepressants | 15,097 (4.8) | 1,513 (12.3) |
| **N02A** | Opioids | 645 (5.0) | 171 (17.1) |  | **A02B** | GI drugs^a^ | 14,964 (4.7) | 2,734 (22.2) |
| **N05A** | Antipsychotics | 555 (4.3) | 30 (3.0) |  | **A10B** | Blood glucose lowering drugs, excl. Insulins | 14,592 (4.6) | 1,664 (13.5) |
| **M01A** | Anti-inflammatory and antirheumatic products, non-steroids | 449 (3.5) | 133 (13.3) |  | **C07A** | Beta blocking agents | 13,365 (4.2) | 2,064 (16.7) |
| **H03A** | Thyroid preparations | 344 (2.6) | 54 (5.4) |  | **N03A** | Antiepileptics | 10,689 (3.4) | 874 (7.1) |
| **N05B** | Anxiolytics | 340 (2.6) | 78 (7.8) |  | **N02A** | Opioids | 9,602 (3.0) | 2,262 (18.3) |
| **C05A** | Agents for treatment of hemorrhoids and anal fissures for topical use | 275 (2.1) | 119 (11.9) |  | **H03A** | Thyroid preparations | 9,081 (2.9) | 1,283 (10.4) |
| **A06A** | Drugs for constipation | 250 (1.9) | 40 (4.0) |  | **B01A** | Antithrombotic agents | 8,210 (2.6) | 1,208 (9.8) |
| **C09A** | Angiotensin-converting enzyme inhibitors, plain | 247 (1.9) | 38 (3.8) |  | **C08C** | Selective calcium channel blockers with mainly vascular effects | 8,104 (2.6) | 1,499 (12.2) |
| **N05C** | Hypnotics and sedatives | 207 (1.6) | 51 (5.1) |  | **C03A** | Low-ceiling diuretics, thiazides | 7,828 (2.5) | 1,698 (13.8) |
| **C10A** | Lipid modifying agents, plain | 199 (1.5) | 44 (4.4) |  | **N02B** | Other analgesics and antipyretics | 7,195 (2.3) | 673 (5.5) |
| **J01M** | Quinolone antibacterials | 199 (1.5) | 138 (13.8) |  | **C09C** | Angiotensin II antagonists, plain | 6,668 (2.1) | 1,196 (9.7) |
| **J01X** | Other antibacterials | 185 (1.4) | 146 (14.6) |  | **N05A** | Antipsychotics | 6,510 (2.1) | 339 (2.8) |
| **G03A** | Hormonal contraceptives for systemic use | 181 (1.4) | 49 (4.9) |  | **C03C** | High-ceiling diuretics | 5,777 (1.8) | 761 (6.2) |
| **N07B** | Drugs used in addictive disorders | 174 (1.3) | 30 (3.0) |  | **N05C** | Hypnotics and sedatives | 5,427 (1.7) | 1,125 (9.1) |
| **R03A** | Adrenergics, inhalants | 172 (1.3) | 61 (6.1) |  | **R03A** | Adrenergics, inhalants | 5,375 (1.7) | 1,191 (9.7) |
| **B03A** | Iron preparations | 157 (1.2) | 35 (3.5) |  | **N05B** | Anxiolytics | 4,558 (1.4) | 1,129 (9.2) |
| **A10B** | Blood glucose lowering drugs, excl. Insulins | 154 (1.2) | 26 (2.6) |  | **A10A** | Insulins and analogues | 3,847 (1.2) | 424 (3.4) |
| **J01C** | Beta-lactam antibacterials, penicillins | 141 (1.1) | 113 (11.3) |  | **B03A** | Iron preparations | 3,688 (1.2) | 624 (5.1) |
| **A10A** | Insulins and analogues | 132 (1.0) | 14 (1.4) |  | **G04C** | Drugs used in benign prostatic hypertrophy | 3,610 (1.1) | 704 (5.7) |
| **L04A** | Immunosuppressants | 130 (1.0) | 17 (1.7) |  | **R03B** | Other drugs for obstructive airway diseases, inhalants | 3,539 (1.1) | 832 (6.7) |
| **--** | **--** | **--** | **--** |  | **M01A** | Anti-inflammatory and antirheumatic products, non-steroids | 3,430 (1.1) | 1,389 (11.3) |
| **--** | **--** | **--** | **--** |  | **M05B** | Drugs affecting bone structure and mineralization | 3,368 (1.1) | 614 (5.0) |
| **--** | **--** | **--** | **--** |  | **A11C** | Vitamin A and D, incl. Combinations of the two | 3,340 (1.1) | 197 (1.6) |
| **--** | **--** | **--** | **--** |  | **S01E** | Antiglaucoma preparations and miotics | 3,207 (1.0) | 516 (4.2) |
| ^a^Gastrointestinal (GI) system drugs: drugs for peptic ulcer and gastro-oesophageal reflux disease. | | | | | | | | |
